# Adaptation and Validation of the Malay Version of the Osteoarthritis Knee and Hip Quality of Life Questionnaire among Knee Osteoarthritis Patients

**DOI:** 10.1155/2018/4329751

**Published:** 2018-05-31

**Authors:** Azidah Abdul Kadir, Mohd Faizal Mohd Arif, Azlina Ishak, Intan Idiana Hassan, Norhayati Mohd Noor

**Affiliations:** ^1^Department of Family Medicine, School of Medical Sciences, Universiti Sains Malaysia, Health Campus, 16150 Kubang Kerian, Kelantan, Malaysia; ^2^Department of Nursing, School of Medical Sciences, Universiti Sains Malaysia, Health Campus, 16150 Kubang Kerian, Kelantan, Malaysia

## Abstract

**Objective:**

To adapt and validate the Malay version of Osteoarthritis Knee and Hip Quality of Life (OAKHQOL) questionnaire.

**Design:**

The OAKHQOL was adapted into Malay version using forward-backward translation methodology. It was then validated in a cross-sectional study of 191 patients with knee osteoarthritis (OA). Patients completed the OAKHQOL and Western Ontario and McMaster Universities Osteoarthritis Index (WOMAC) questionnaire. Confirmatory analysis, reliability analysis, and Pearson correlation test were performed.

**Results:**

The new five-factor model of 28 items demonstrated an acceptable level of goodness of fit (comparative fit index = 0.915, Tucker-Lewis index = 0.905, incremental fit index = 0.916, chi-squared/degree of freedom = 1.953, and root mean square error of approximation = 0.071), signifying a fit model. The Cronbach's alpha value and the composite reliability of each construct ranged from 0.865 to 0.933 and 0.819 to 0.921, respectively. The Pearson correlation coefficient between the OAKHQOL and the WOMAC showed adequate criterion validity. Known groups validity showed statistical difference in body mass index in physical activity, mental health, and pain construct. The pain domain was statistically different between the age groups.

**Conclusion:**

The Malay version OAKHQOL questionnaire is a valid and reliable instrument to assess health-related quality of life in knee OA patients.

## 1. Introduction

Osteoarthritis (OA) is the most common disease of joints in adults around the world [1, 2]. Due to its chronicity in nature, it is the major cause of pain and disability. OA may affect not only physical functioning, but also mental health (anxiety and depression), sleep, work ability, interpersonal interactions, self-esteem, quality of life, sexuality, and participation [3, 4].

There are few validated instruments used in studies to assess health-related QOL in patients with OA specifically. The Medical Outcomes Study Short-Form 36 (SF36), which has been widely applied to assess QOL, is not disease-specific to OA and was found to have low response rate in population more than 65 years of age [5]. The Lequesne index and Western Ontario and McMaster Universities Osteoarthritis (WOMAC) questionnaire, which are more disease-specific, are able to measure only pain and function but not the other domains of QOL such as mental, social, and sexual domains [6, 7]. It was suggested that the SF-36 and WOMAC should be used in combination [8]; however, they may still fail to capture specific QOL aspects related to hip or knee osteoarthritis. Knee Injury and Osteoarthritis Outcome Score (KOOS) is another questionnaire but its assessment is not limited to quality of life because it includes pain, other symptoms, activity of daily living, sports, and recreational activity measurements [9]. Thus, the Osteoarthritis Knee and Hip Quality of Life (OAKHQOL) scale questionnaire was developed and validated to measure the impact of specifically knee and hip osteoarthritis on the patient's QOL.

The OAKHQOL is a specific tool to measure QOL in knee and hip OA as it takes into account specific themes that are exclusive to the QOL of patients with knee and hip OA (social support, sleep, side effects of drugs, plans for the future, embarrassment to be seen by people, use of public transport, difficulty in moving after staying in the same position, and sexuality) [10]. It has 43 items which fall into five domains: physical activity, pain, mental health, social functioning, and social support. Evaluation of the OAKHQOL has shown the reliability of the five domains to be satisfactory (interclass correlation coefficients: 0.70–0.85), the construct validity to be adequate (Spearman correlation coefficients: 0.43–0.75), and the discrimination to be satisfactory [10].

Confirmatory factor analysis (CFA) is a theory-testing model as opposed to exploratory factor analysis (EFA) which is a theory-generating method [11]. CFA is a type of structural equation modeling (SEM) that specifically deals with measurement models, that is, the relationships between observed measures or indicators and latent variables or factors [12, 13]. It is powerful because it provides explicit hypothesis testing for factor analytic problems.

Assessing the QOL among knee OA patients is important to ensure holistic care for the patient. Despite this, the reliability and validity of the OAKHQOL in the Malaysian context have not been established. The need for a validated questionnaire suitable to the local population based on the language is very important as it is more accurate to illustrate the real impact of the disease on the patient's QOL. Hence, this study aimed to determine the psychometric properties of the Malay version of the OAKHQOL among knee OA patients.

## 2. Materials and Methods

A cross-sectional study was conducted among 191 patients diagnosed with knee OA between February and August 2014 at the Outpatient Clinic, Universiti Sains Malaysia Hospital, a tertiary teaching hospital in Malaysia. A total of 210 patients were invited, and only 191 patients fulfilled the inclusion and exclusion criteria and were recruited in the study, which give the response rate of 90%. Patients with unilateral or bilateral knee osteoarthritis diagnosed according to the clinical and radiological criteria of the American College of Rheumatology (knee pain and radiographic osteophytes plus at least one of three symptoms/signs), aged more than 50 years, who experienced morning stiffness of less than 30 minutes and crepitus on active motion, and who were able to read in the Malay language were included.

Convenience sampling was applied, and written informed consent was taken. Patients were asked to fill out the Malay version OAKHQOL (Table 7) and the validated Malay Version WOMAC. Sociodemographic data (age, gender, education, and race) and knee OA history were taken. Body mass index (BMI) measurements and weight-bearing anterior-posterior view X-rays of both knees were taken. The participants took about 15 minutes to complete both questionnaires. They also did not have to pay for their participation in the study.

Sample size was determined based on Hair et al. (2010). The minimum sample size required for five or less constructs was 100 samples [14].

### 2.1. OAKHQOL Questionnaire

This questionnaire was developed by Rat et al. to assess quality of life in knee and hip OA patients [10, 15], specifically to assess health-related quality of life (HRQOL) [10]. The concept of this questionnaire was based on the World Health Organization (WHO) definition of QOL. This is a self-administered questionnaire. The original questionnaire was developed in French and later in English [15]. It was shown to capture patients' perceptions of their disease, and it possesses the necessary psychometric properties of validity and reliability for use in clinical trials and observational studies [10, 15].

In the original validation study, four factors were identified in the exploratory factor analysis based on scree plot and Eigen values. These factors were physical activities (19 items), mental health (14 items), social support (four items), and social functioning (three items). The pain factor (four items) was found to have loaded on the physical and mental health factors. However, based on expert opinions, the pain factor was included as an individual dimension. Therefore, the final English version of OAKHQOL consists of 43 items divided into five dimensions: physical activity, mental health, pain, social support, and social functioning as well as three additional items [10]. The three additional items are relationship, sexual activity, and professional life. The five dimensions (40 items) (Table 8) and three additional items are intended to be used separately. The three additional items are independent items and were not included in the analysis. Each item in the five dimensions is measured on a numerical rating scale from 0 to 10. The final scores were the mean of scores of all the items in respective domains that ranged between 0 to 10 [10].

### 2.2. Adaptation of the OAKHQOL Questionnaire

Forward and backward translation was carried out by a group of panelists consisting of family medicine specialists, physicians, linguists, and bilingual laymen. Modifications were made, and content validity was checked. The revised version was tested on 20 patients for face validity. These patients were excluded from the psychometric analysis.

### 2.3. WOMAC

WOMAC is a disease-specific, self-administered health status measure that is widely used to assess the symptoms and physical disability for people with hip and/or knee OA [16, 17]. It is widely used in OA research especially to evaluate clinical outcome measures as a result of treatment intervention [18]. The WOMAC measures total pain score, total stiffness score, and total physical functioning score. The original index consists of 24 questions (five questions for pain, two questions for stiffness, and 17 questions for physical function). It has been validated in Bahasa, Malaysia [16]. This questionnaire is available in a Likert version rated on an ordinal scale of 0 to 4 and also as a visual analog scale (VAS) [16]. In this study, a VAS version was used.

### 2.4. Statistical Analysis

Confirmatory factor analysis (CFA), reliability analysis, and Pearson correlation test were performed to assess the psychometric properties using SPSS version 22.0 and Analysis of Moment Structure (AMOS) software version 21.0. On preliminary data screening, cases with incomplete response were removed from data. Further assessment of normality and outliers was performed on the factor scores based on the critical ratio (i.e., for skewness and kurtosis to their standard error) and the Mahalanobis distance. Mahalanobis distance was used to identify the outliers by using AMOS software. It computes and tabulates the distance of every data from the center of all data distribution [19].

The CFA was performed to examine the goodness of fit indices of the Malay OAKHQOL latent construct. Construct validity examines the degree to which a scale measures what it intends to measure [20]. Construct validity is achieved if the goodness of fit indices signify a model fit [20].

The measurement of the model fit was checked with several goodness of fit indicators: comparative fit index (CFI), Tucker-Lewis index (TLI), incremental fit index (IFI), chi-squared/degree of freedom, and root mean square error of approximation (RMSEA) [12, 13, 21]. For approximate fit index, a value of more than 0.9 was taken for CFI, IFI, and TLI [21, 22]. Chi-squared/degree of freedom of less than 3 and RMSEA value of less than 0.08 were taken as indicators of an acceptable level [12, 13, 19].

In addition to the overall evaluation of goodness of fit, the standardized factor loading (standardized regression weight) modification indices (MI) and squared multiple correlation (*R*^2^) were used as indicators to select which items should be removed in the model [12, 13]. MI suggested correlations between variables. A high MI value indicates redundancy in a pair of variables [12, 13]. Discriminant validity is also assessed by obtaining correlation values between the constructs. A correlation of more than 0.85 between constructs is considered to indicate poor discriminant validity [12, 13].

Reliability analysis was measured using Cronbach's alpha coefficient, composite reliability (CR), and average variance extracted (AVE). Reliability refers to the accuracy and precision of the measurement procedure. Cronbach's alpha coefficient was measured using SPSS. Both CR and AVE were derived from CFA analysis and manually calculated based on published formula [19, 23]. A Cronbach's alpha coefficient value of more than 0.7 and a CR equal to or greater than 0.6 represent a measure of satisfactory internal consistency [19, 24, 25]. AVE is the average percentage of variation explained by the variables in the construct or domain. The acceptable value for it was taken as more than 0.5 [19]. In this study, the test-retest reliability was not done due to time constraint and limited budget.

The Pearson correlation test was performed to assess the criterion validity of the OAKHQOL. The test was done between the pain construct of the OAKHQOL and the pain construct of the WOMAC, as well as between the physical construct of the OAKHQOL and the functional construct of the WOMAC. The correlation coefficient of more than 0.5 but less than 0.8 was considered to be a good correlation [26].

Known group validity is a method to support construct validity of a questionnaire. The method will evaluate the questionnaire ability to discriminate between the two groups known to differ on the variable of interest [27]. In this study, known group validity was assessed through gender, BMI, age groups of the patients, and Kellgren–Lawrence grading of the knee radiograph. We hypothesized that females, those aged more than 60 years [28], those having a BMI greater than 25 kg/m^2^, and those with more severe radiographic grading would have significant differences [10]. An independent *t*-test was used to analyze for gender, BMI, and age groups of the patients. One-way ANOVA test was used to analyze radiographic grading based on Kellgren–Lawrence classification. In the analysis for known group validity, the score for each domain was normalized to 0–100.

## 3. Results

### 3.1. Translation and Cultural Adaptation

We found that all the items in the Malay version questionnaire are relevant and appropriate to the Malaysian population. All the items were found to be acceptable, clear, and easy to understand in the face validity.

### 3.2. Psychometric Properties

A total of 191 patients participated in the study. The sociodemographic and knee OA disease characteristics of the participants are shown in Table 1. The mean age was 57.8 (6.8) years and the majority were female. The Kellgren–Lawrence classification ranged from grade 0 to 4.

### 3.3. Descriptive Statistics of the Items

The items in the five constructs of OAKHQOL had missing data ranging from 0 to 3 values (0%–1.5%). However, the individual items concerning professional life, relationships, and sexual activities had 10 to 13 missing values. The missing values were replaced with the mean scores for the domain during the CFA. Normality assessment was done for the 40 items in the five constructs using histogram, box-plot, and measurement of skewness, which showed normal distribution. The absolute and percentage frequencies of the score for all the items were calculated and illustrated in Table 2.

### 3.4. Confirmatory Analysis

Confirmatory factor analysis was performed with one-step strategy. Confirmatory analysis showed that the original five-factor model of the OAKHQOL (40 items) was not fit (Table 3). Five items (py25, m36, m37, m38, and m16) were removed one by one due to low factor loadings, as shown in Model A. Eight items were set as free parameter estimates, one pair at a time (py1-py2, py7-py8, py4-py5, and pn34-pn33), based on high MI (greater than 15) as shown in Model C. Further item deletion was done based on MI and factor loadings (py3, py9, m29, py24, sp39, py14, and py13) until the final model, which consists of a five factors with 28 items, signified a model fit (Table 3). The final model consists of five constructs: physical activity (10 items), mental health (eight items), social functioning (three items), social support (three items), and pain (four items). Six items in the physical activity, five items in the mental health, and one item in the social support were removed. The goodness of fit indices indicated that the model had a good construct (CFI = 0.915, TLI = 0.905, IFI = 0.916, chi-squared/degree of freedom = 1.953, and RMSEA = 0.071) (Table 3).

**Figure 1:**
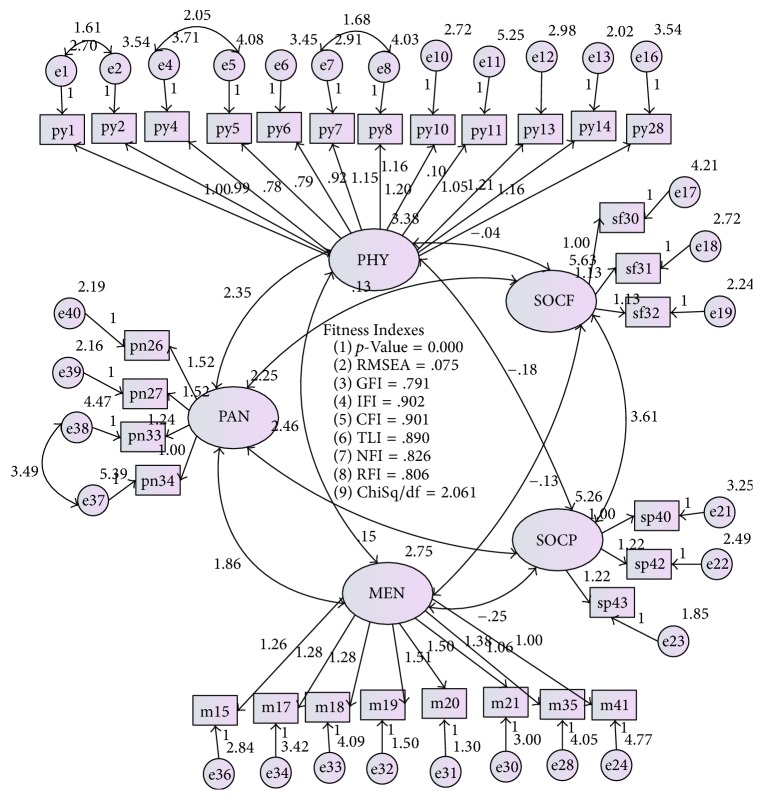
The initial AMOS graphic shows the goodness of fit indexes, respective path coefficient, factor loading, and *R*2. PHY: physical activity, SOCF: social functioning, SOCP: social support, MEN: mental health, PAN: pain.

**Figure 2:**
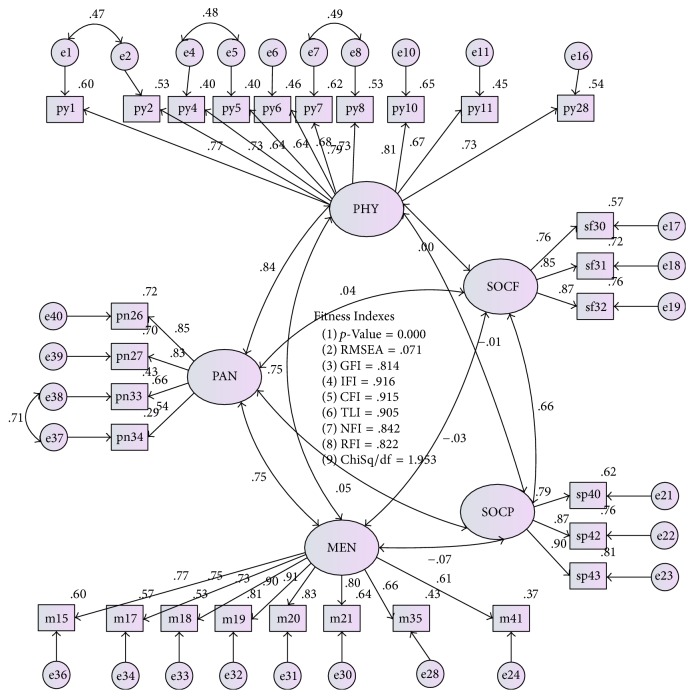
The AMOS graphic shows the goodness of fit indexes, respective path coefficient, factor loading, and *R*2. The final model shows 5 constructs and 28 items. PHY: physical activity, SOCF: social functioning, SOCP: social support, MEN: mental health, PAN: pain.

The initial model before fit was shown in Figure 1. The correlation between factors was illustrated in Figure 2. The standardized factor loadings were from 0.5 to 0.9, indicating that all items contributed highly to the construct measures. The MI values were less than 10, and the correlation between each pair of latent constructs was less than 0.85, which is acceptable (Figure 2) [19].

### 3.5. Reliability

The reliability analysis showed that the Cronbach's alpha coefficient value for each construct was greater than 0.7 (Table 4). The CR and AVE of each construct also showed that the final construct had a good measure of reliability. The result was achieved by using one-step estimation strategy.

Table 5 shows the Pearson's correlation coefficients between the physical activity construct of the OAKHQOL and the functional construct of the WOMAC (*r* = 0.72) and between the pain construct of the OAKHQOL and pain construct of the WOMAC (*r* = 0.55). These results indicated that the OAKHQOL had acceptable criterion validity.

### 3.6. Known Group Validity

The results for the known group validity of the OAKHQOL are shown in Table 6. We found significant differences among the BMI groups (BMI ≤ 25 kg/m^2^ and >25 kg/m^2^) in the physical activity (*p* = 0.009), mental (*p* = 0.040), and pain domains (*p* = 0.009). We also found significant differences among the groups based on OA severity according to radiographic grading in the physical activity (*p* = 0.002) and pain domains (*p* = 0.043). Thus, groups who had greater disease severity based on radiography had worse scores. The scores of the pain domain for the age groups (age ≤ 60 years compared to those age > 60 years) were also significant. There were no differences observed for the social support and social function domains.

## 4. Discussion

Recently, validated health-related quality of life that accurately reflects a patient's experience with respect to specific disease has been an important outcome recommended for interventional study. Health-related quality of life is a broad concept representing individual responses to physical, mental, and social effects on daily living. Therefore, the need to assess conceptual relevance and psychometric properties in various cultures or countries is increasing [15].

The present study indicated that the shortened Malay version of the OAKHQOL had good validity and reliability and is culturally acceptable. EFA of the original OAKHQOL using principle component analysis with orthogonal varimax rotation revealed four factors: physical activities, mental health, social support, and social functioning with the pain factor as an individual dimension [10, 15]. The OAKHQOL has also been validated in Spanish and Persian [2, 29]. However, to our knowledge, this is the first study that used confirmatory analysis in the validation analysis. CFA is used to verify the factor structure of a measurement instrument. CFA has become more commonly used for construct validation and to provide evidence for convergent and discriminant validity of the theoretical construct [30]. Furthermore, CFA is a theory-testing model and it starts with a hypothesis prior to the analysis which is based on strong theoretical and/or empirical foundation [31]. On the other hand, EFA is used to explore the possible underlying factor structure of a measurement instrument [32].

The panel in this study decided to keep the original five-factor model in the initial analysis, although the EFA of the original study did not support this. EFA of the original study was done in other language; thus the result was different. The decision to keep the pain construct in the final model was made because we found the pain factor to be an important domain that is also available in and consistent with other health-related QOL for OA measures and the items were also culturally acceptable [16, 17].

We made the decision to remove six items from the physical activity construct (py3, py9, py13, py14, py24, and py25), five items in the mental health construct (m16, m29, m36, m37, and m38), and one item in the social support construct (sp39) because other items in the construct reflected similar functions. Most of the items were removed because of significant overlapping (high modification indices) and lack of discrimination within the items. Removal of these items was shown to improve the fit indices of the model, indicating that perhaps they poorly represented the construct being measured. However, the panel of this study had also revisited and reviewed the items before they were removed because they might represent important and meaningful construct as mentioned in a previous validation study.

The reliability analysis showed that internal consistency of the Malay version OAKHQOL was acceptable. Other than that, the CR and AVE for each construct were also acceptable, indicating that they had good levels of internal consistency. As for the criterion validity, the analysis showed that the physical and pain constructs of the Malay version of OAKHQOL had good correlations with the functional and pain constructs of the WOMAC. In Malay version of OAKHQOL, physical activity construct has 10 items whereas WOMAC has 17 items in functional construct [16]. In physical activity and functional construct of both questionnaires, daily activities such as difficulty in walking, bending, going up and down the stairs, and getting in and out of a car or a bus were assessed. Physical activity on self-care such as taking bath, getting dressed, and cutting toe-nails was assessed in OAKHQOL, whereas WOMAC assessed other aspects of daily activities such as difficulty in sitting, standing, lying on bed, getting up from sitting or from bed, shopping, and also doing house chores [16]. Perhaps future research can examine the criterion validity for the mental construct, social functioning, and social support of the Malay version of OAKHQOL. The SF36 is one questionnaire that has been used to assess health-related QOL for people with knee OA, although it is not disease-specific. This questionnaire has been validated in the Malay language. We suggest correlating the OAKHQOL scores with the SF36 in a future study.

For the known group validity, we found that the Malay version of OAKHQOL discriminates well for the BMI groups and the severity of disease based on plain radiograph for the physical activity, pain, and mental domains. However, for the social domains, it was not discriminative based on disease severity. This finding was similar to the findings of De Tejada et al., who conducted the validation study in Spanish [2]. Both Malay and English versions after validation are shown in Tables 9 and 10.

This study is not without limitation. First, this study involved only people with knee OA; therefore, the findings may not be generalized to patients with hip OA. In addition, the convenient sampling was applied. Thus, it may not represent the true knee OA population in the community. It is also good to measure the responsiveness of this questionnaire in a clinical trial where it can be used to evaluate changes in patient status following therapeutic intervention.

## 5. Conclusion

The Malay version of OAKHQOL consisting of five factors assessed through 28 items was valid, reliable, and acceptable to measure quality of life in Malaysian population with knee OA.

## Figures and Tables

**Table 1:** Sociodemographic and clinical characteristics of knee OA patients.

Variables	Mean	(SD)	*N* (%)
Age (year)	57.8	6.8	
Gender:			
Male			63 (33)
Female			128 (67)
Education:			
Primary			30 (15.7)
Secondary			132 (69.1)
Tertiary			29 (15.2)
Race:			
Malay			186 (97.4)
Chinese			5 (2.6)
BMI (kg/m^2^)	28.5	5.1	
Duration of knee OA (year)	3.7	3.7	
Knee joint affected			
Left			43 (22.5)
Right			34 (17.8)
Both			114 (59.7)
Kellgren–Lawrence classification:			
0			59 (30.9)
1			68 (35.6)
2			33 (17.3)
3			27 (14.1)
4			4 (2.1)

**Table 2:** Absolute and percentage frequencies of score for all items.

Score item		0	1	2	3	4	5	6	7	8	9	10	Total
Py1	Freq	11	24	20	24	26	38	12	20	7	3	6	191
	%	5.8	12.6	10.5	12.6	13.6	19.9	6.3	10.5	3.7	1.6	3.1	100
Py2	Freq	9	14	25	23	15	37	17	21	13	10	7	191
	%	4.7	7.3	13.1	12.0	7.9	19.4	8.9	11.0	6.8	5.2	3.7	100
Py3	Freq	11	15	16	12	23	32	25	19	16	15	7	191
	%	5.8	7.9	8.4	6.3	12.0	16.8	13.1	9.9	8.4	7.9	3.7	100
Py4	Freq	8	10	11	21	22	45	20	23	15	12	4	191
	%	4.2	5.2	5.8	11.0	11.5	23.6	10.5	12.0	7.9	6.3	2.1	100
Py5	Freq	7	8	12	15	20	23	32	33	20	15	6	191
	%	3.7	4.2	6.3	7.9	10.5	12.0	16.8	17.3	10.5	7.9	3.1	100
Py6	Freq	49	32	25	23	14	22	9	7	4	4	2	191
	%	25.7	16.8	13.1	12.0	7.3	11.5	4.7	3.7	2.1	2.1	1.0	100
Py7	Freq	35	22	21	16	20	26	22	11	10	7	1	191
	%	18.3	11.5	11.0	8.4	10.5	13.6	11.5	5.8	5.2	3.7	0.5	100
Py8	Freq	35	24	18	16	14	28	16	11	15	9	5	191
	%	18.3	12.6	9.4	8.4	7.3	14.7	8.4	5.8	7.9	4.7	2.6	100
Py9	Freq	2	13	17	11	20	32	28	21	17	23	7	191
	%	1.0	6.8	8.9	5.8	10.5	16.8	14.7	11.0	8.9	12.0	3.7	100
Py10	Freq	18	14	19	16	17	36	27	16	12	10	6	191
	%	9.4	7.3	9.9	8.4	8.9	18.8	14.1	8.4	6.3	5.2	3.1	100
Py11	Freq	33	15	14	14	31	25	14	12	12	7	4	191
	%	17.3	7.9	7.3	7.3	16.2	13.1	7.3	6.3	6.3	3.7	7.3	100
Py13	Freq	45	24	23	18	17	27	17	13	2	2	3	191
	%	23.6	12.6	12.0	9.4	8.9	14.1	8.9	6.8	1.1	1.1	1.6	100
Py14	Freq	31	17	22	20	21	32	16	15	11	3	3	191
	%	16.2	8.9	11.5	10.5	11.0	16.8	8.4	7.9	5.8	1.6	1.6	100
M15	Freq	25	19	21	19	22	29	23	13	8	9	3	191
	%	13.1	9.9	11.0	9.9	11.5	15.2	12.0	6.8	4.2	4.7	1.6	100
M16	Freq	20	21	19	18	14	27	25	20	13	7	7	191
	%	10.5	11.0	9.9	9.4	7.3	14.1	13.1	10.5	6.8	3.7	3.7	100
M17	Freq	18	26	21	16	17	26	20	18	16	8	5	191
	%	9.4	13.6	11.0	8.4	8.9	13.6	10.5	9.4	8.4	4.2	2.6	100
M18	Freq	40	20	18	20	17	22	16	12	15	7	4	191
	%	20.9	10.5	9.4	10.5	8.9	11.5	8.4	6.3	7.9	3.7	2.1	100
M19	Freq	36	28	18	15	18	29	17	12	8	7	3	191
	%	18.8	14.7	9.4	7.9	9.4	15.2	8.9	6.3	4.2	3.7	1.6	100
M20	Freq	33	27	22	17	16	34	15	9	6	9	3	191
	%	17.3	14.1	11.5	8.9	8.4	17.8	7.9	4.7	3.1	4.7	1.6	100
M21	Freq	51	27	13	22	7	29	11	13	9	7	2	191
	%	26.7	14.1	6.8	11.5	3.7	15.2	5.8	6.8	4.7	3.7	1.0	100
Py24	Freq	6	11	19	19	18	27	27	19	22	15	8	191
	%	3.1	5.8	9.9	9.9	9.4	14.1	14.1	9.9	11.5	7.9	4.2	100
Py25	Freq	100	21	16	10	6	14	4	5	3	4	8	191
	%	52.4	11.0	8.4	5.2	3.1	7.3	2.1	2.6	1.6	2.1	4.2	100
Pn26	Freq	9	16	23	24	20	30	13	21	15	12	8	191
	%	4.7	8.4	12.0	12.6	10.5	15.7	6.8	11.0	7.9	6.3	4.2	100
Pn27	Freq	20	14	25	25	16	31	18	16	14	6	6	191
	%	10.5	7.3	13.1	13.1	8.4	16.2	9.4	8.4	7.3	3.1	3.1	100
Py28	Freq	46	31	15	18	12	27	13	12	7	7	3	191
	%	24.1	16.2	7.9	9.4	6.3	14.1	6.8	6.3	3.7	3.7	1.6	100
M29	Freq	39	24	18	17	18	30	22	4	9	8	2	191
	%	20.4	12.6	9.4	8.9	9.4	15.7	11.5	2.1	4.7	4.2	1.0	100
Sf30	Freq	20	8	24	17	14	34	15	8	14	16	21	191
	%	10.5	4.2	12.6	8.9	7.3	17.8	7.9	4.2	7.3	8.4	11.0	100
Sf31	Freq	17	12	18	20	14	36	18	6	7	19	24	191
	%	8.9	6.3	9.4	10.5	7.3	18.8	9.4	3.1	3.7	9.9	12.6	100
Sf32	Freq	12	12	12	19	24	28	16	9	15	18	26	191
	%	6.3	6.3	6.3	9.9	12.6	14.7	8.4	4.7	7.9	9.4	13.6	100
Pn33	Freq	44	24	21	13	12	32	16	9	14	4	2	191
	%	23.0	12.6	11.0	6.8	6.3	16.8	8.4	4.7	7.3	2.1	1.0	100
Pn34	Freq	51	24	21	11	17	27	13	10	13	3	1	191
	%	26.7	12.6	11.0	5.8	8.9	14.1	6.8	5.2	6.8	1.6	0.5	100
M35	Freq	19	33	30	17	13	37	11	12	8	6	5	191
	%	9.9	17.3	15.7	8.9	6.8	19.4	5.8	6.3	4.2	3.1	2.6	100
M36	Freq	40	29	23	17	20	30	11	7	9	4	1	191
	%	20.9	15.2	12.0	8.9	10.5	15.7	5.8	3.7	4.7	2.1	0.5	100
M37	Freq	68	29	19	15	15	26	7	4	3	5	0	191
	%	35.6	15.2	9.9	7.9	7.9	13.6	3.7	2.1	1.6	2.6	0	100
M38	Freq	37	28	20	18	17	34	12	9	9	6	1	191
	%	19.4	14.7	10.5	9.4	8.9	17.8	6.3	4.7	4.7	3.1	5	100
Sp39	Freq	9	11	12	16	14	23	17	23	16	16	34	191
	%	4.7	5.8	6.3	8.4	7.3	12.0	8.9	12.0	8.4	8.4	17.8	100
Sp40	Freq	9	9	15	11	13	39	14	12	26	21	22	191
	%	4.7	4.7	7.9	5.8	6.8	20.4	7.3	6.3	13.6	11.0	11.5	100
M41	Freq	45	24	22	17	11	37	13	6	6	7	3	191
	%	23.6	12.6	11.5	8.9	5.8	19.4	6.8	3.1	3.1	3.7	1.6	100
sp42	Freq	10	6	16	12	9	23	13	16	14	20	52	191
	%	5.2	3.1	8.4	6.3	4.7	12.0	6.8	8.4	7.3	10.5	27.2	100
sp43	Freq	11	10	13	15	12	28	16	15	19	18	34	191
	%	5.8	5.2	6.8	7.9	6.3	14.7	8.4	7.9	9.9	9.4	17.8	100

Py: physical activity; m: mental health; pn: pain; sf: social functioning; sp: social support.

**Table 3:** Fitness level of models.

5-factor model	RMSEA	CFI	IFI	TLI	Chi Square/df	Actions taken
Original: (40 item)	0.100	0.770	0.772	0.754	2.908	

Model A: 35 items	0.094	0.826	0.827	0.811	2.680	Delete
						py25, m36, m37, m38
						m16

Model B: 35 items	0.094	0.843	0.844	0.829	2.680	Correlate between the errors
						py7-py8
						py4-py5
						py1-py2
						pn33-pn34

Model C 30 items	0.090	0.901	0.902	0.890	2.061	Delete: py3, py9,
						m29, py24, sp 39

Final model: 28 items	0.071	0.915	0.916	0.905	1.953	Delete py14 and py13

CFI: comparative fit index; TLI: Tucker-Lewis index; IFI: incremental fit index; RMSEA: root mean squared error of approximation.

**Table 4:** Reliability and confirmatory factor analysis of the Malay version OAKHQOL.

Construct	Item	Factor loading	Cronbach alpha	CR	AVE
Physical activity	py1	0.774	0.933	0.915	0.743
	py2	0.736			
	py4	0.653			
	py5	0.646			
	py6	0.672			
	py7	0.771			
	py8	0.716			
	py10	0.804			
	py11	0.679			
	py28	0.742			

Mental	m15	0.773	0.919	0.921	0.796
	m17	0.754			
	m18	0.726			
	m19	0.899			
	m20	0.910			
	m21	0.798			
	m35	0.656			
	m41	0.608			

Social functioning	sf30	0.756	0.865	0.867	0.867
	sf31	0.851			
	sf32	0.871			

Social support	sp40	0.786	0.888	0.890	0.686
	sp42	0.871			
	sp43	0.901			

Pain	pn34	0.538	0.809	0.819	0.540
	pn33	0.657			
	pn27	0.834			
	pn26	0.847			

CR: construct reliability; AVE: average variance extracted.

**Table 5:** Pearson correlation coefficient.

	*r*	*p* value
Physical activity domain OAKHQOL and functional domain WOMAC	0.72	<0.001
Pain domain WOMAC and pain domain OAKHQOL	0.55	<0.001

Correlation is significant at the 0.05 level (2-tailed).

**Table 6:** Known group validity of the Malay version OAKHQOL.

Variables		Physical			Mental			Pain			Social functioning			Social support		
	*N*	Mean	SD	*p* value	Mean	SD	*p* value	Mean	SD	*p* value	Mean	SD	*p* value	Mean	SD	*p* value
Gender:				0.208			0.370			0.890			0.761			0.328
Male	63	37.5	17.7		33.8	22.4		37.7	20.9		49.9	29.2		57.4	27.4	
Female	128	41.8	21.5		36.7	22.2		38.2	23.5		51.2	27.4		61.5	28.3	
Age:				0.321			0.208			**0.025**			0.767			0.929
≤60 years	161	39.7	19.9		34.8	22.2		36.7	21.9		51.1	28.2		60.3	27.9	
>60 years	30	43.6	22.2		40.1	22.5		45.1	25.5		49.2	26.9		59.8	29.2	
BMI				**0.009**			**0.040**			**0.009**			0.434			0.940
<25	50	32.5			30.2			30.9	20.2		53.5	29.3		59.9	28.6	
≥25	141	42.3			37.7			40.5	22.9		49.9	27.4		60.2	27.9	
KL grading				**0.002**			0.125			**0.043**			0.527			0.424
0	59	37.8	18.1		33.6	22.4		38.4	22.9		47.6	30.1		58.1	27.9	
1	68	36.4	19.2		32.0	20.7		32.6	21.1		51.2	29.3		59.9	29.5	
2	33	42.7	21.1		41.2	21.1		39.3	20.8		57.5	21.9		64.9	24.9	
3	27	50.0	22.6		41.7	26.0		47.5	24.7		47.7	26.4		56.7	28.9	
4	4	69.3	13.5		46.3	13.3		50.0	29.8		56.7	24.6		80.9	13.4	

BMI: body mass index (kg/m^2^), KL: Kellgren–Lawrence grading of knee X-ray.

**Table 7:** Prevalidation of Malay version of OAKHQOL questionnaire (40 items).

		Domain
1	Saya mengalami kesusahan untuk berjalan	py1
2	Saya mengalami kesusahan untuk tunduk atau meluruskan badan/bangun semula	py2
3	Saya mengalami kesusahan untuk membawa barang yang berat	py3
4	Saya mengalami kesusahan untuk menuruni tangga	py4
5	Saya mengalami kesusahan untuk menaiki tangga	py5
6	Saya mengalami kesusahan untuk mandi	py6
7	Saya mengalami kesusahan untuk berpakaian lengkap (seperti memakai stoking, kasut, seluar dan sebagainya)	py7
8	Saya mengalami kesusahan untuk memotong kuku kaki	py8
9	Saya mengalami kesusahan untuk bergerak selepas lama berada dalam kedudukan yang sama	py9
10	Saya mengalami kesusahan untuk masuk dan keluar daripada kereta	py10
11	Saya mengalami kesusahan untuk menggunakan kenderaan awam (bas, teksi dsb)	py11
12	Saya perlukan masa untuk bersendiri/saya perlu bersendiri	py13
13	Saya memerlukan masa yang lebih lama untuk melakukan sesuatu perkara	py14
14	Saya kurang bersemangat disebabkan sakit	m15
15	Saya risau jika saya perlu bergantung kepada orang lain	m16
16	Saya risau menjadi tidak berkemampuan	m17
17	Saya merasa malu apabila orang melihat saya	m18
18	Saya berasa gelisah	m19
19	Saya berasa tertekan	m20
20	Saya merasakan kehidupan keluarga saya terjejas	m21
21	Saya mengalami kesusahan untuk berada dikedudukan yang sama untuk jangkamasa yang lama (duduk, berdiri, tidak bergerak dsb)	py24
22	Saya memerlukan tongkat atau alat bantu untuk berjalan	py25
23	Saya mengalami kesakitan (kekerapan)	pn26
24	Saya mengalami kesakitan (keterukan)	pn27
25	Saya memerlukan pertolongan untuk membuat sesuatu seperti kerja rumah dan membeli belah	py28
26	Saya rasa lebih tua daripada umur saya	m29
27	Saya mampu/boleh merancang projek/program untuk jangkamasa yang panjang	sf30
28	Saya keluar rumah sekerap mana yang saya suka	sf31
29	Saya melayan tetamu di rumah sebanyak mana yang saya suka	sf32
30	Saya mengalami kesukaran untuk tidur atau tidur semula kerana sakit	pn33
31	Saya terjaga disebabkan sakit	pn34
32	Saya tertanya-tanya apa yang bakal berlaku/kan terjadi kepada saya	m35
33	Saya pemarah/mudah marah atau agresif	m36
34	Saya rasa saya menyakiti hati mereka yang rapat dengan saya	m37
35	Saya merasa risau tentang kesan sampingan rawatan saya	m38
36	Saya boleh berkongsi dengan orang lain tentang kesukaran yang saya alami disebabkan penyakit sendi (arthritis) sebanyak mana yang saya suka	sp39
37	Saya merasakan orang lain faham tentang kesusahan yang saya alami disebabkan penyakit sendi (arthritis) saya	sp40
38	Saya merasa malu untuk meminta bantuan/pertolongan jika perlu	m41
39	Saya rasa saya diberi sokongan oleh orang yang rapat dengan saya (pasangan dan keluarga)	sp42
40	Saya rasa saya diberi sokongan oleh orang yang berada di sekeliling saya (kawan dan jiran)	sp43

Py: physical activity; m: mental; pn: pain; sf: social functioning; sp: social support.

**Table 8:** Prevalidation of English version of OAKHQOL questionnaire (40 items).

		Domain
1	I have difficulty walking	py1
2	I have difficulty bending down or straightening up	py2
3	I have difficulty carrying heavy things	py3
4	I have difficulty going down stairs	py4
5	I have difficulty climbing stairs	py5
6	I have difficulty taking a bath	py6
7	I have difficulty getting dressed	py7
8	I have difficulty cutting my toe-nails	py8
9	I have difficulty getting going again after staying in the same position for a long time	py9
10	I have difficulty getting in and out of a car	py10
11	I have difficulty using public transport	py11
12	I have to pace myself	py13
13	I take more time to do things	py14
14	My spirits are low because of the pain	m15
15	I worry about being dependent on others	m16
16	I worry about being disabled	m17
17	I feel embarrassed when people look at me	m18
18	I am anxious	m19
19	I am depressed	m20
20	I feel my family life is being affected	m21
21	I have difficulty staying in the same position for a long time	py24
22	I need a walking stick/cane or crutches to walk	py25
23	I have pain (describe frequency)	pn26
24	I have pain (describe intensity)	pn27
25	I need help for things like housework and shopping	py28
26	I feel older than my age	m29
27	I am able to plan projects for the long term	sf30
28	I get out of the house as much as I like	sf31
29	I entertain at home as much as I like	sf32
30	I have difficulty getting to sleep or getting back to sleep because of pain	pn33
31	I wake up because of pain	pn34
32	I wonder what will become of me	m35
33	I am irritable or aggressive	m36
34	I feel I annoy those close to me	m37
35	I am worried about the side effects of my treatment	m38
36	I can talk to others about the difficulties I have due to my arthritis as much as I like	sp39
37	I feel others understand the difficulties I have because of my arthritis	sp40
38	I am embarrassed to ask for help if I need it	m41
39	I feel supported by people close to me	sp42
40	I feel supported by those around me	sp43

Py: physical activity; m: mental; pn: pain; sf: social functioning; sp: social support.

**Table 9:** Postvalidation of English version of OAKHQOL questionnaire (28 items).

		Domain
1	I have difficulty walking	py1
2	I have difficulty bending down or straightening up	py2
3	I have difficulty going down stairs	py4
4	I have difficulty climbing stairs	py5
5	I have difficulty taking a bath	py6
6	I have difficulty getting dressed	py7
7	I have difficulty cutting my toe-nails	py8
8	I have difficulty getting in and out of a car	py10
9	I have difficulty using public transport	py11
10	My spirits are low because of the pain	m15
11	I worry about being disabled	m17
12	I feel embarrassed when people look at me	m18
13	I am anxious	m19
14	I am depressed	m20
15	I feel my family life is being affected	m21
16	I have pain (describe frequency)	pn26
17	I have pain (describe intensity)	pn27
18	I need help for things like housework and shopping	py28
19	I am able to plan projects for the long term	sf30
20	I get out of the house as much as I like	sf31
21	I entertain at home as much as I like	sf32
22	I have difficulty getting to sleep or getting back to sleep because of pain	pn33
23	I wake up because of pain	pn34
24	I wonder what will become of me	m35
25	I feel others understand the difficulties I have because of my arthritis	sp40
26	I am embarrassed to ask for help if I need it	m41
27	I feel supported by people close to me	sp42
28	I feel supported by those around me	sp43

Py: physical activity; m: mental; pn: pain; sf: social functioning; sp: social support.

**Table 10:** Postvalidation of Malay version of OAKHQOL questionnaire (28 items).

		Domain
1	Saya mengalami kesusahan untuk berjalan	py1
2	Saya mengalami kesusahan untuk tunduk atau meluruskan badan/bangun semula	py2
3	Saya mengalami kesusahan untuk menuruni tangga	py4
4	Saya mengalami kesusahan untuk menaiki tangga	py5
5	Saya mengalami kesusahan untuk mandi	py6
6	Saya mengalami kesusahan untuk berpakaian lengkap (seperti memakai stoking, kasut, seluar dan sebagainya)	py7
7	Saya mengalami kesusahan untuk memotong kuku kaki	py8
8	Saya mengalami kesusahan untuk masuk dan keluar daripada kereta	py10
9	Saya mengalami kesusahan untuk menggunakan kenderaan awam (bas, teksi dsb)	py11
10	Saya kurang bersemangat disebabkan sakit	m15
11	Saya risau menjadi tidak berkemampuan	m17
12	Saya merasa malu apabila orang melihat saya	m18
13	Saya berasa gelisah	m19
14	Saya berasa tertekan	m20
15	Saya merasakan kehidupan keluarga saya terjejas	m21
16	Saya mengalami kesakitan (kekerapan)	pn26
17	Saya mengalami kesakitan (keterukan)	pn27
18	Saya memerlukan pertolongan untuk membuat sesuatu seperti kerja rumah dan membeli belah	py28
19	Saya mampu/boleh merancang projek/program untuk jangkamasa yang panjang	sf30
20	Saya keluar rumah sekerap mana yang saya suka	sf31
21	Saya melayan tetamu di rumah sebanyak mana yang saya suka	sf32
22	Saya mengalami kesukaran untuk tidur atau tidur semula kerana sakit	pn33
23	Saya terjaga disebabkan sakit	pn34
24	Saya tertanya-tanya apa yang bakal berlaku/kan terjadi kepada saya	m35
25	Saya merasakan orang lain faham tentang kesusahan yang saya alami disebabkan penyakit sendi (arthritis) saya	sp40
26	Saya merasa malu untuk meminta bantuan/pertolongan jika perlu	m41
27	Saya rasa saya diberi sokongan oleh orang yang rapat dengan saya (pasangan dan keluarga)	sp42
28	Saya rasa saya diberi sokongan oleh orang yang berada di sekeliling saya (kawan dan jiran)	sp43

Py: physical activity; m: mental; pn: pain; sf: social functioning; sp: social support.
